# Monitoring trends in the absolute lymphocyte count and the neutrophil-to-lymphocyte ratio in patients with breast cancer receiving eribulin

**DOI:** 10.1186/s12885-024-11923-5

**Published:** 2024-02-12

**Authors:** Meng-Xia Su, Hsiang-Wen Lin, Hanh T. H. Nguyen, Tien-Chao Lin, Chih-Jung Chen, Hwei-Chung Wang, Chen-Teng Wu, Yao-Chung Wu, Geng-Yan He, Liang-Chih Liu, Chih-Hao Huang

**Affiliations:** 1 Surgical Department, China Medical University Hospital, No. 2, Yude Rd. North Dist, Taichung, 404327 Taiwan; 2 School of Pharmacy and Graduate Institute, China Medical University, No. 100, Sec. 1, Jingmao Rd., Taichung, 406040 Taiwan; 3 Department of Clinical Pharmacy, Hanoi University of Pharmacy, No. 144, Xuan Thuy, Cau giay, Hanoi, Vietnam; 4 Department of Pharmacy, China Medical University Hospital, No. 2, Yude Rd., North Dist., Taichung, 404327, Taiwan; 5 Department of Pharmacy System, Outcomes and Policy, College of Pharmacy, University of Illinois at Chicago, Chicago, 833 S. Wood St., Chicago, 60612 Illinois United States of America; 6 College of Medicine, China Medical University, No. 100, Sec. 1, Jingmao Rd., Taichung, 406040 Taiwan

**Keywords:** Breast cancer, Eribulin, Absolute lymphocyte count, Neutrophil-to-lymphocyte count

## Abstract

**Background:**

Studies have shown that the absolute lymphocyte count (ALC) and the neutrophil-to-lymphocyte ratio (NLR) are related to the outcomes in patients with breast cancer receiving specific chemotherapies. However, the reports have focussed on the initial blood test and there is a lack of evidence or data to support that dynamic changes of ALC or NLR are associated with the patients’ survival outcomes.

**Methods:**

We retrospectively reviewed electronic medical records from patients with breast cancer treated with eribulin from 2015 to 2019 at our institution. Blood test data were available prior to starting eribulin (baseline), and at 1, 3 and 6 months after initiating eribulin. We classified the patients into ALC and NLR high and low groups using the following cut-offs: 1000/µl for ALC and 3 for NLR. We defined ALC and NLR trends as increasing or decreasing compared with the initial data. We assessed the associations between the ALC and NLR with progression-free survival and overall survival.

**Results:**

There were 136 patients with breast cancer treated with eribulin. Of these patients, 60 had complete blood tests and follow-up data. Neither a high ALC nor a low baseline NLR was associated with the survival outcome. One month after initiating eribulin treatment, a high ALC and a low NLR were significantly associated with longer progression-free survival (*p* = 0.044 for each). Three months after initiating eribulin, a high ALC was significantly associated with better overall survival (*p* = 0.006). A high NLR at 3 or 6 months after initiating eribulin was associated with worse overall survival (*p* = 0.017 and *p* = 0.001, respectively). The ALC and NLR trends across times were not associated with survivals.

**Conclusion:**

We showed that 1, 3 and 6 months after initiating eribulin, a high ALC and a low NLR may be related to the patients’ survival outcomes. The ALC and NLR trends were not associated with survival. Accordingly, we believe patients who maintain a high ALC and a low NLR may have better clinical outcomes after initiating eribulin.

**Supplementary Information:**

The online version contains supplementary material available at 10.1186/s12885-024-11923-5.

## Background

Cytotoxic chemotherapy is still a mainstay of treatment for recurrent advanced or metastatic breast cancer (A/MBC) because most of the patients are usually treated with chemotherapy sooner or later. Although some hormone receptor-positive patients first receive endocrine therapy, most of them eventually would need chemotherapy as well [1]. Eribulin, a novel chemotherapeutic agent, was licensed for the treatment of A/MBC in patients who had previously received at least one chemotherapy regimen in the metastatic settings. It improved overall survival (OS) in women with A/MBC in the phase III EMBRACE clinical trial [2]. To date, there is no consensus regarding the use of biomarkers across times to predict whether a patient with A/MBC would benefit from eribulin or not.

Inflammation plays an important role in cancer development and responses to therapies, so researchers have evaluated the ability of inflammatory biomarkers in blood to predict the survival outcomes of patients with various types of cancers and receiving various treatments, including patients with A/MBC receiving eribulin [3–11]. They have examined the ability of the absolute lymphocyte count (ALC) and the neutrophil-to-lymphocyte ratio (NLR) - which can be measured easily and inexpensively - to predict the cancer prognosis [12–17]. Eribulin exerts an anticancer effect by inhibiting microtubule dynamics, and it is thought to have biological effects on the immune system by suppressing the epithelial-to-mesenchymal transition (EMT) and vascular remodelling and by improving the tumour microenvironment. These actions contribute to an improvement in OS in patients with A/MBC [18]. Several studies have shown that a high ALC or a low baseline NLR is associated with better survival outcomes in patients with A/MBC treated with eribulin [4, 5, 7, 8], but not for patients receiving traditional treatments [3, 19].

Almost all of available studies have focussed mainly on the ALC or NLR values at the baseline, i.e., before the initiation of a treatment, to identify patients who might benefit from monitoring these measures [3–5, 7, 8, 19]. Some researchers suggest that the associated factors could change dynamically during treatment with several therapies. For example, researchers have noted a significant increase in the ALC of patients with human epidermal growth factor receptor 2 (HER2)-positive A/MBC after one cycle of trastuzumab emtansine treatment, or dissimilar changes in the ALC and NLR during the treatment with eribulin and bevacizumab among patients with A/MBC or non-small cell lung cancer, respectively [6, 20, 21]. Such changes might reflect a patient’s real-time response to the treatments, or could be used to predict a patient’s prognosis more precisely [6, 20, 21]. Moreover, Nakamoto et al. [6] demonstrated that dynamic changes in ALC after one cycle of treatment with eribulin seemed to be an independent predictor for post-progress survival among patients with A/MBC, even when eribulin was discontinued. In this study, we examined the ability of the ALC and NLR at different times after eribulin had been initiated to predict progression-free survival (PFS) and OS of patients with A/MBC. We hypothesise that the changes in the ALC and NLR after eribulin initiation allow clinicians to predict a patient’s outcomes regardless of whether the patient is still receiving eribulin or not.

## Methods

### Patients

For this retrospective cohort study, data were retrieved from the electronic medical records (eMRs) of patients with A/MBC treated with eribulin at China Medical University Hospital (CMUH) between 1 January 2015 and 30 June 2019. Female patients with locally recurrent or metastatic breast cancer, age ≥ 20 years and who received at least one cycle of eribulin for A/MBC were included. Patients with a second cancer or who did not have complete blood counts at the initiation of eribulin treatment or at 1, 3 and 6 months following eribulin initiation were excluded for further analysis.

### Treatment with eribulin

Eribulin was administered intravenously at a dose of 1.4 mg/m^2^ on days 1 and 8 of each 21-day cycle. The physician could adjust the eribulin dose based on the severity of adverse events. The treatment was continued until the disease being progressed or encounter intolerable of toxicity.

### ALC and NLR

This study focussed on the effect of eribulin after its initiation irrespective of whether it was continued or discontinued. Complete blood counts -including white blood cells, neutrophils and lymphocytes - were evaluated at four time points, which were determined based on the time since the eribulin initiation date (the index date). Practically, the lab data closest to the defined time points (within 2 weeks) were used for analysis. The first time point is baseline, just prior to the initial eribulin dose. At CMUH, a complete blood count is usually obtained at the beginning of each planed treatment cycle as part of the routine clinical practice. The subsequent three time points were at 1, 3 and 6 months after eribulin initiation. The NLR was calculated by dividing the number of neutrophils by the number of lymphocytes. The ALC and NLR were classified as high or low using a cut-off of 1000/µl for the ALC and 3 for the NLR; these values were chosen based on the available studies [5, 14, 22, 23]. The trends in the ALC and NLR 1, 3 and 6 months after eribulin initiation were categorised as decreasing if the ALC and NLR were lower compared with baseline and non-decreasing (including increasing) if they were equal or higher compared with baseline.

### Outcomes

The primary outcome was PFS: the time from initial eribulin treatment until disease progression or death. If eribulin was discontinued due to treatment intolerance or any other reasons with no reported disease progression, then the patient was censored. The secondary outcome was OS, which was computed from eribulin initiation until death. Considering CMUH is a tertiary hospital with comprehensive cancer diagnosis and treatment facilities in middle of Taiwan, we assumed those patients who were lost to follow-up at CMUH might be due to either they decided to stop receiving further treatment in their end stages of cancer or they had encountered the competing risks for death with no records in CMUH. In practice, some patients might choose to either receive home hospice care or die at home with CMUH discharge medical records indicated “discharge against medical advice” for their end-stage care. Some might be transferred to the other medical institutes based on patients’ preferences or special needs of out-of-pocket treatments, which might be not available in CMUH. In this case, these patients were censored because we assumed they were not treated continuously at CMUH or until the study observation period had ended. Consequently, these patients were treated as death events at the time of loss to follow-up to obtain a stricter estimate of OS.

### Statistical analysis

The patient characteristics are presented with descriptive statistics, including the median and interquartile range (IQR) and frequencies. Kaplan-Meier curves were generated to assess the trends in PFS and OS at each time point based on the ALC and NLR groups. The log-rank test was used to compare the survival outcomes between the groups. A univariate Cox proportional-hazards regression model was used to explore the factors associated with the ALC and NLR at different time points and the ALC and NLR trends and PFS and OS. Those factors associated with PFS and OS (with *p* ≤ 0.15) were used to adjust for the associations of the ALC, NLR and their trends with PFS and OS in the multivariate Cox proportional hazards regression model. When analysing the ALC and NLR trends, the baseline values were also used as a covariate for adjustment. When comparing the ALC and NLR 1, 3 and 6 months after eribulin initiation with the baseline ALC and NLR, whether the patient was still receiving eribulin treatment at the corresponding time points, except for some specific concerns, was also considered an important covariate for multivariate Cox regression. Statistical significance was set at *p* < 0.05. All statistical analyses were performed with SAS 9.4 (SAS Institute, Cary, NC, USA).

## Results

There were 136 patients with A/MBC who were prescribed with eribulin at CMUH from January 2015 to June 2019. We excluded patients without baseline or follow-up tests, who did not complete at least one cycle of eribulin or who did not have a sufficient follow-up time. Finally, we included 60 patients in the study. Their median age was 51 (IQR 41-59) years (Table 1). Of the patients, 57.6%, 50.0% and 37.3% were oestrogen receptor (ER) positive, progesterone receptor (PR) positive and HER2 positive, respectively, and 16.7% of them were diagnosed with triple-negative breast cancer. The majority of the patients had metastasis at two or more sites (66.7%). Moreover, 60% of the patients experienced visceral metastasis (15.0% visceral and 45.0% visceral and non-visceral metastasis). In terms of treatment features, 78.4% of all assessed patients had previously been treated with chemotherapy as adjuvant or neo-adjuvant therapy. One fourth of the patients received eribulin as first- or second-line chemotherapy, and three fourths received eribulin as third-line and higher chemotherapy for their advance disease. The median chemotherapy line of eribulin was 4 (IQR 3-5). The majority of patients (76.7%) received eribulin as monotherapy.

**Table 1:** Characteristics among assessed advanced or metastatic breast cancer breast cancer (A/MBC) patients treated with Eribulin (*N*=60)

**Characteristics**	**N**	**%**
Age (years): Median (IQR)	51 (41 – 59)	
Estrogen receptor^a^ (*N*=59)		
Positive	34	57.6
Negative	25	42.4
Progesterone receptor^a^ (*N*=58)		
Positive	29	50.0
Negative	29	50.0
HER2 status^a^ (*N*=59)		
Negative	37	62.7
Positive	22	37.3
Triple negative breast cancer		
No	50	83.3
Yes	10	16.7
Number of metastatic sites: Median (IQR)	2 (1-3)	
Number of metastatic sites:		
<2 sites (0 or 1)	20	33.3
≥2 sites	40	66.7
Types of metastasis:		
Non-visceral	24	40.0
Visceral	27	45.0
Both visceral and non-visceral	9	15.0
(Neo)adjuvant chemotherapy^a^ (*N*=37)		
No	8	21.6
Yes	29	78.4
Eribulin used line to treat recurrent or metastatic breast cancer: Median (IQR)	4 (3 - 6)	
Early-line (First and second line)	15	25.0
Late-line (≥ 3^rd^ line)	45	75.0
Eribulin duration (days): Median (IQR)	123 (68 – 185)	
Eribulin treated		
As monotherapy	46	76.7
With Trastuzumab (±pertuzumab)	6	10.0
With Hormone therapy	5	8.3
With Others^b^	3	5.0

*IQR* Interquartile range, *HER2* Human epidermal growth factor receptor 2, *TNBC* Triple negative breast cancer

^a^the calculation was based on those patients with available data accordingly

^b^others include those were prescribed with pembrolizumab, bevacizumab, capecitabin

In univariate Cox regression analysis, none of the patient demographic and clinical characteristics were significantly associated with PFS (Table 2). However, patients with an older age, more than one metastatic site and third-line or higher eribulin treatment were at increased risk of mortality (i.e. OS) compared with younger patients (hazard ratio [HR] 1.04, 95% confidence interval [CI] 1.01-1.07), those with one or no metastatic sites (HR 2.50, 95% CI 1.02-6.13) and those receiving first- or second-line eribulin (HR 4.30, 95% CI 1.30-14.22). The HRs for PFS considering patients who still used eribulin at 3 or 6 months after eribulin initiation were 4.43 (95% CI 2.11-9.30) and 4.04 (95% CI 1.97-8.30), respectively. These values are consistent with the expected PFS for the patients with A/MBC treated with eribulin and not included in multivariate analysis.

**Table 2:** Factors associated with progression-free-survival (PFS) and overall survival (OS) among A/MBC patients treated with Eribulin (univariate cox proportional-hazards analysis)

	**PFS**		**OS**	
	**HR (95%CI)**	***p***	**HR (95%CI)**	***p***
**Demographic and clinical characteristics**				
Age (years)	1.00 (0.97 – 1.03)	0.778	1.04 (1.00 – 1.07)	**0.032**
Estrogen receptor (Negative vs. Positive)	1.12 (0.59 – 2.14)	0.727	0.87 (0.41 – 1.83)	0.714
Progesterone receptor (Negative vs. Positive)	1.30 (0.68 – 2.46)	0.426	0.74 (0.36 – 1.55)	0.430
HER2 status (Positive vs. Negative)	1.22 (0.63 – 2.37)	0.557	0.88 (0.43 – 1.82)	0.732
TNBC (Yes vs. No)	0.97 (0.40 – 2.34)	0.947	1.12 (0.39 – 3.22)	0.840
Number of metastatic site (> 1 vs. ≤1)	1.77 (0.82 – 3.82)	**0.150**	2.50 (1.02 -6.13)	**0.045**
Metastatic sites				
Non visceral metastasis	1		1	
Visceral and bone (2)	1.24 (0.61 – 2.54)	0.550	1.79 (0.81 – 4.00)	0.152
Visceral only (3)	1.60 (0.57 – 4.49)	0.389	1.28 (0.43 – 3.81)	0.658
Eribulin line of therapy for recurrent and metastatic breast cancer (≥3^rd^ vs. <3^rd^)	1.62 (0.76 – 3.46)	0.212	4.30 (1.30 – 14.22)	**0.017**
(Neo)adjuvant (No vs. Yes)	1.37 (0.53 – 3.49)	0.516	0.95 (0.34 – 2.64)	0.925
Monotherapy of eribulin (No vs. Yes)	0.97 (0.46 – 2.07)	0.945	1.03 (0.46 -2.32)	0.941
**Still use eribulin or not at the time point of test as compare with that at baseline**				
Off/on eribulin treatment at 1-month-test	0.99 (0.23 – 4.34)	0.988	4.21 (0.89 – 19.82)	**0.069**
Off/on eribulin treatment at 3-month-test	4.43 (2.11 – 9.30)	**<0.0001**	0.99 (0.45 – 2.15)	0.973
Off/on eribulin treatment at 6-month-test	4.04 (1.97 – 8.30)	**<0.0001**	1.921 (0.88 – 4.21)	0.103

*A/MBC* Advanced or metastatic breast cancer, *PFS* Progression-free-survival, *OS* Overall survival

**Fig. 1:**
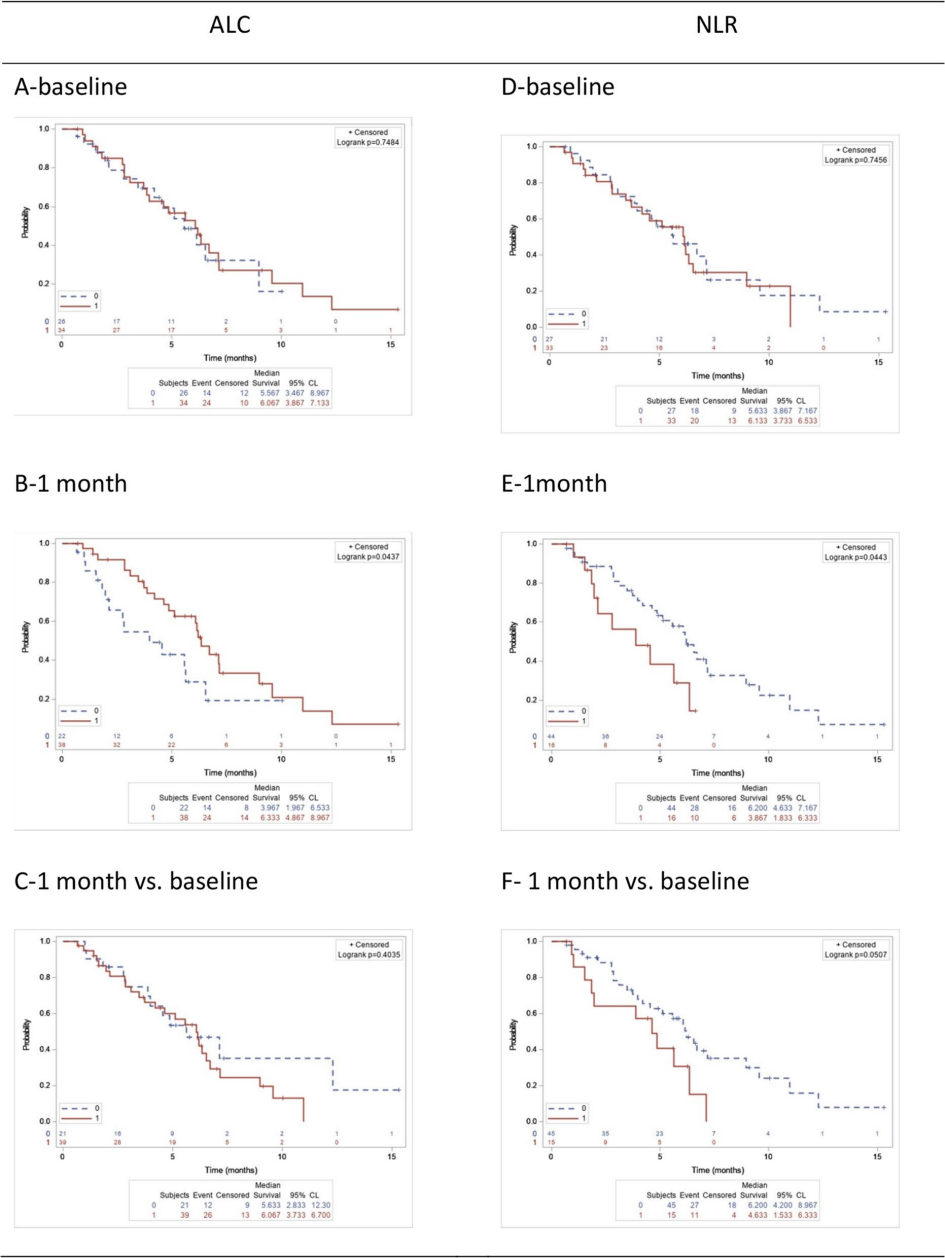
Progression-free survival (PFS) among patients with high and low ALC and NLR

Kaplan-Meier analysis followed by the log-rank test revealed no association between a high ALC or a low NLR at baseline with PFS (all *p* > 0.05, Fig. 1A and D). In contrast, 1 month after eribulin initiation, a high ALC and a low NLR were significantly associated with longer PFS compared with a low ALC (*p* = 0.044, Fig. 1B) or a high NLR (*p* = 0.044, Fig. 1E). The ALC trend was not associated with PFS (*p* = 0.403, Fig. 1C). Moreover, a decreasing trend for NLR at 1 month after eribulin initiation showed no significant association with better PFS compared with a non-decreasing trend (*p* = 0.051, Fig. 1F).

**Fig. 2:**
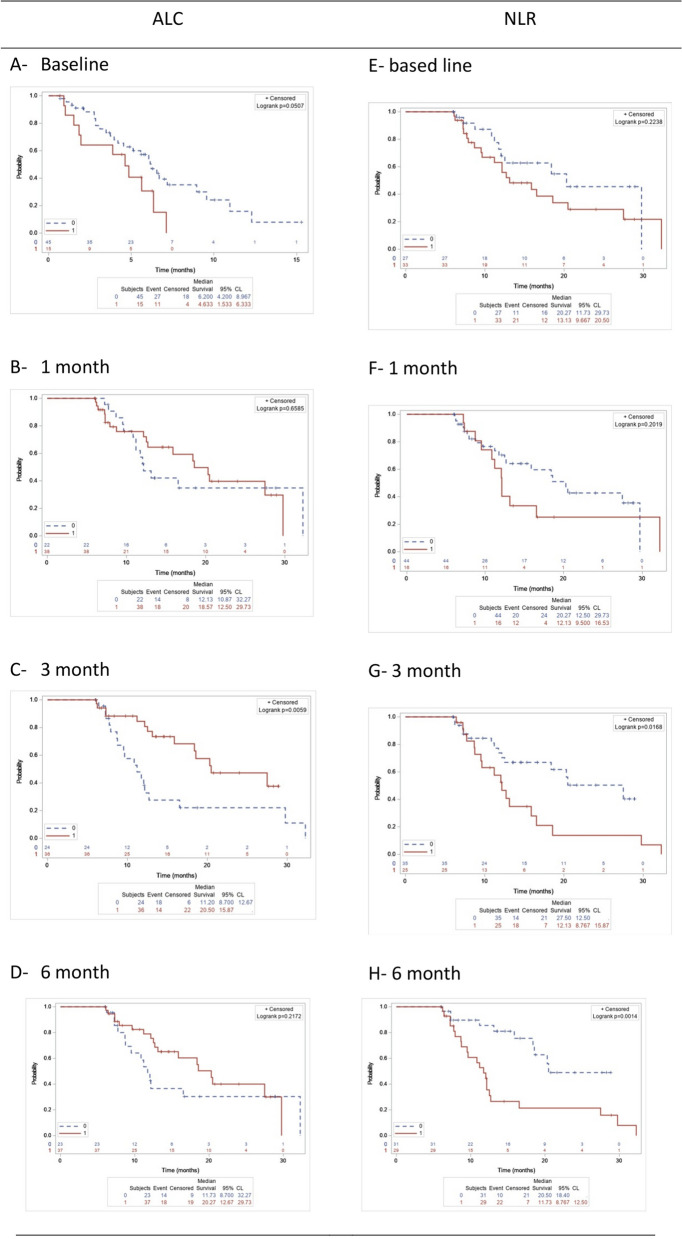
Overall survival (OS) among patients with high and low ALC and NLR. ALC= absolute lymphocyte count; NLR= neutrophil to lymphocyte ratio; OS= Overall survival; 0=low; 1=high in (**A**) (**B**) (**C**) (**D**) (**E**) (**F**) (**G**) (**H**). OS of patients with high and low ALC at the baseline (**A**), 1month (**B**), 3-month (**C**) and 6-month test (**D**); OS of patients with high and low NLR at the baseline (**E**), 1- month (**F**), 3-month (**G**) and 6-month test (**H**)

The trends of OS among patients with high and low ALC and NLR at 1-, 3-,6-month tests were listed in Fig. 2A-2H, accordingly. In particular, a high ALC at baseline or 3 months after eribulin initiation was significantly associated with better OS compared with a low ALC (*p* = 0.011 and *p* = 0.006 respectively, Fig. 2A and C). A high NLR at 3 or 6 months after eribulin initiation was significantly associated with worse OS compared with a low NLR (*p* = 0.017 and *p* = 0.001, respectively, Fig. 2G and H). The ALC and NLR trends at all the time points did not show an association with OS (Supplementary Fig. 1). However, there were significant differences among the groups of patients stratified by ALC trends according to the baseline ALC level (Supplementary Fig. 2), and among different groups of patients stratified by the NLR baseline level and the NLR trend at 6 months after eribulin initiation compared with baseline (Supplementary Fig. 3).

In the multivariate Cox regression analysis, only the ALC at 1 month after eribulin initiation was significantly associated with PFS. Specifically, patients with a low ALC had 2.26 times (95% CI 1.12-4.55) higher risk of disease progression after adjusting for the number of metastatic sites and off/on treatment with eribulin (Table 3). For OS, a low ALC at the baseline was significantly associated with a higher risk of mortality than a high baseline ALC (HR 2.89, 95% CI 1.33-6.26). Patients with a low ALC or a high NLR at 3 months after eribulin initiation had a higher risk of mortality compared with those with a high ALC (HR 2.30, 95% CI 1.03-5.11) or a low NLR (HR 2.37, 95% CI 1.07-5.24). After adjusting for the confounding factors, a non-decreasing trend for the NLR at 6 months after eribulin initiation was significantly associated with a higher risk of mortality compared with a decreasing trend (HR 6.12, 95% CI 1.83-20.45). We also considered the associations between OS and a high versus low NLR/ALC stratified by on/off treatment with eribulin at 3 or 6 months after eribulin initiation. There was a significant association between OS and the NLR at 3 and 6 months after eribulin initiation, and a significant association between OS and the ALC at 3 months after eribulin initiation (Supplementary Fig. 4).

**Table 3:** Associations between ALC, NLR, their trends and progression-free-survival (PFS) or overall survival (OS) among patients used Eribulin using multivariate cox proportional-hazards analysis

	PFS^a^		OS^a^	
	HR (95%CI)	*p*	HR (95%CI)	*p*
ALC level (Low vs.High [ref]) ^b^				
At baseline	1.05 (0.53 – 2.08)	0.888	2.89 (1.33 – 6.26)	0.007*
At 1-month-test^c^	2.26 (1.12 – 4.55)	0.023*	1.33 (0.63 – 2.83)	0.462
At 3-month-test^c^	-	**-**	2.30 (1.03 – 5.11)	0.042*
At 6-month-test^c^	-	-	1.14 (0.54 – 2.40)	0.728
ALC trends (Decreasing vs. Non-decreasing[ref])				
1-month-test compared with the baseline^d,c^	0.82 (0.38 – 1.75)	0.608	0.88 (0.38 – 2.04)	0.768
3-month-test compared with the baseline^d,c^	-	**-**	2.08 (0.88 – 4.94)	0.095
6-month-test compared with the baseline^d,c^	-	-	0.76 (0.34 – 1.73)	0.515
NLR level (High vs. Low[ref])^e^				
At baseline	0.98 (0.50 – 1.91)	0.944	1.59 (0.74 – 3.43)	0.240
At 1-month-test^c^	1.97 (0.96 – 4.27)	0.087	1.45 (0.67 – 3.14)	0.344
At 3-month-test^c^	-	-	2.37 (1.07 – 5.24)	0.033*
At 6-month-test^c^	-	-	2.06 (0.85 – 5.00)	0.109
NLR trends (Non-decreasing vs. non-decreasing [ref])				
1-month-test compared with the baseline^f,c^	2.18 (0.92 – 5.18)	0.078	2.15 (0.86 – 5.39)	0.103
3-month-test compared with the baseline^fc^	-	-	2.50 (0.92 – 6.85)	0.074
6-month-test compared with the baseline^f,c^	-	-	6.12 (1.83 – 20.45)	0.003*

*HR* Hazard ratio, *CI* Confidence interval, *ALC* Absolute lymphocyte count, *PFS* Progression-free-survival, *OS* Overall survival

^a^Adjusted for the factors have *p*≤0.15 in the univariate analysis above (number of metastatic sites for PFS; number of metastatic sites, eribulin line of treatment and age for OS).

^b^ALC high: ≥1000/µl, low: <1000/µl

^*^p<0.05

^c^Additionally, model was adjusted for off/on eribulin treatment at time point corresponding with the tests

^d^Additionally, model was adjusted for ALC at baseline

^e^NLR high: ≥3, low: <3

^f^Additionally, model was adjusted for NLR at baseline

## Discussion

To our knowledge, this is the first study to explore the changes in the ALC and NLR across times and proved blood test results in different timing after initiating eribulin treatment. The changes in the ALC and NLR are important predictors for PFS and OS among patients with A/MBC. Specifically, a high ALC or a low NLR at 1 month after eribulin initiation was significantly associated with better PFS in patients with A/MBC. Moreover, a high ALC at 1 month after eribulin initiation was an independent predictor of PFS after controlling for the other factors. After controlling for the other factors, a high baseline ALC, a low NLR at 3 months after eribulin initiation or a non-decreasing NLR trend at 6 months after eribulin initiation were the independent factors associated with extended OS in patients with A/MBC.

After initiating eribulin treatment, the ALC status in patients with A/MBC could be a reliable predictor of the outcomes, with a high ALC being favourable. The ALC in the peripheral blood is considered as a possible indicator of the tumour immune microenvironment [3] and has well-known anti-tumour properties [24]. Moreover, eribulin can alter the tumour immune microenvironment by suppressing the EMT and vascular remodelling [18, 25, 26].

We found that an ALC > 1000/µl at 1 month after eribulin initiation was significantly associated with longer PFS than an ALC < 1000/µl. There have been a few studies in which researchers investigated the associations between ALC changes and clinical outcomes, and the available findings are inconsistent [6, 21]. Imamura et al. [21] reported that a significant increase in the ALC and a non-significant change in the neutrophil count following one cycle of trastuzumab emtansine treatment for patients with A/MBC. However, a significant decrease in the NLR (i.e. a significant increase in the ALC and a non-significant change in the neutrophil count) was significantly associated with improved PFS. Nakamoto et al. showed that a decrease in the ALC after one cycle with eribulin was linked to a longer time to treatment failure [6]. In contrast, we did not find an association between the ALC trend and PFS. However, the findings support the idea that a high ALC at 1 month after eribulin initiation was associated with improved PFS. Based on our study, we believe the ALC change at 1 month after eribulin initiation could be a potential predictor of better PFS among patients treated with eribulin. However, further cross-validation studies in different medical institutes are needed to confirm our findings.

In the post hoc analysis of the EMBRACE phase III clinical trial, the baseline ALC was a specific predictor of OS in eribulin-treated patients [3]. Specifically, a high baseline ALC was a potential predictor of OS among patients with A/MBC in the eribulin group but not in the control group (other treatments based on physician’s choice). Other studies have reported that a high baseline ALC was associated with improved OS despite the use of different ALC cut-offs (e.g. 1000/µl, 1500/µl or the median ALC value) [4–6, 8]. In the current study, a baseline ALC > 1000/µl was an independent predictor of OS, a finding consistent with previous studies [9, 27–29]. Ideally, this finding could be used to facilitate clinical decision-making to identify patients who may benefit from treatment with eribulin. Although the baseline ALC has been robustly demonstrated to be a specific predictor of OS in patients with A/MBC treated with eribulin, the predictive value of the ALC regarding PFS remains unclear. Some studies did show the significant associations of high baseline ALC and improved PFS [9, 27], but some indicated a non-significant association [28, 29]. In this current study, we did not find an association between the baseline ALC and PFS.

In contrast to the ALC, the baseline NLR does not seem to be a good candidate biomarker to predict the response to eribulin therapy. A few systematic reviews and meta-analyses have indicated the role of baseline NLR as one of predictors for PFS and OS for patients with various types of cancer (including breast cancer) [30–34]. On additional study indicated that baseline NLR was one of predictors of partial response to eribulin but not for PFS among metastatic breast cancer patients [35]. Although the post hoc analysis of the EMBRACE clinical trial showed that the baseline NLR was predictor for OS [3], Corbeau et al’s systematic review did include this aforementioned study and indicted that the NLR was an independent predictor of survival in BC patients among those adjuvant treatment studies but not for but those early BC patients “receiving neo-adjuvant chemotherapy and advanced BC patients [14]. In our study, we did not find a significant correlation between the baseline NLR and PFS or OS. Therefore, we believe the baseline ALC should be considered a more specific predictor for patients treated with eribulin than the baseline NLR.

Nevertheless, changes in the NLR after eribulin initiation could be potential predictors of survival. Regarding PFS, NLR and its trend at 1 month after eribulin initiation was significantly associated with PFS in the Kaplan-Meier analysis. These findings are consistent with another study in which the authors found that a decrease in the NLR after one cycle was associated with improved PFS in patients with A/MBC treated with trastuzumab emtansine [21]. Other studies have revealed that the NLR after one or two chemotherapy cycles could be considered predictors for PFS among patients with advanced colorectal or urothelial cancer [36, 37]. In terms of OS, the dynamic changes in the NLR at 3 and 6 months after eribulin initiation were independent predictors of OS. This finding is consistent with a previous study that indicated an increasing trend in the NLR after six cycles of bevacizumab was linked to a higher mortality risk in patients with advanced non-small cell lung cancer [20]. In this case, we presume that the use of eribulin is associated with a long-term outcome like OS after sufficient follow-up (i.e. 3 months or 6 months after eribulin initiation), only if the effect of eribulin is stable. In contrast, Nakamoto et al. [6] revealed that the dynamic change in the NLR after one cycle or at the end of eribulin treatment among patients with A/MBC did not show a significant relationship with OS.

There are some limitations to this study. First, it was a retrospective single-centre study. As a result, the information used to determine the outcomes was heavily influenced by the local clinical practice. In particular, the definition of death is based on local practice; it includes those who were lost to follow-up and were deemed to be at high risk of dying at the CMUH. However, we applied this definition to all patient groups. Second, this study had a relatively small sample size and the findings might be not able to apply to those patients treated in the other hospitals. We were afraid to encounter type II error due to small sample size to make it unlikely to uncover significant relationships between prognostic factors and outcomes. In other words, the lack of a significant association from the multivariate analysis could be attributable to the small sample size. Further research to recruit more patients (conducting prospective cohort study) and to compare with the patients treated in different institutes or even in different countries might be helpful to confirm the robustness of the findings. Third, we do not know whether the changes in the ALC and NLR are particular predictors for eribulin because the associations obtained from the statistical analysis could not be interpreted as causality. Fourth, we cannot rule out that other factors might have interfered with the blood tests, such as acute infection and glucocorticoid usage, given we performed a retrospective study using electronic medical records. Future prospective research with a greater number of patients across times in different institutes might be able to address these aforementioned limitations.

In conclusion, we have shown that changes in the ALC and NLR following initial eribulin treatment could be potential predictors of PFS and OS in patients with A/MBC. Specifically, a high ALC at 1 month after eribulin initiation was significantly associated with better PFS, whereas a low ALC or a high NLR at 3 months after eribulin initiation or a non-decreasing NLR trend at 6 months were significantly associated with higher a mortality risk as compared with a high ALC, a low NLR or a decreasing NLR, respectively. Thus, the ALC and NLR, which can be measured relatively easily and inexpensively, at 1-, 3-, 6-month seem to be helpful to either identify individuals with A/MBC who could potentially benefit from eribulin, or to predict their responses and prognosis of cancer. Future studies should be conducted simultaneously at different medical institutes, use nationwide databases or perform a prospective study design to understand the impact of using the ALC and NLR across times as predictors of breast cancer survival outcomes.

PFS of patients with high and low ALC at the baseline (A), at the 1-month test (B), and its trends at 1-month test (as compared with baseline) (C); PFS of patients with high and low NLR at the baseline (D), at the 1-month test (E), and its trend at 1-month test (as compared with baseline) (F).

### Supplementary Information

**Additional file 1:** **Supplementary Figure 1.** Overall survival (OS) among patients with increased or decreased trends of ALC, NLR (0: decreasing, 1: non-decreasing). **Supplementary Figure 2.** Comparing PFS and OS among different groups of patients stratified by ALC trends according to the baseline level. **Supplementary Figure 3.** Comparing PFS and OS among different groups of patients stratified by NLR baseline and trends. **Supplementary Figure 4.** Analysis on the association of high(1)/low(0) NLR/ALC with OS stratified by on(1)/off(0) treatment with eribulin.

## Data Availability

The detailed patient databases generated and analyzed during this study are not publicly available due to appropriate protection of patient personal information but are available from the corresponding author on reasonable request.

## Abbreviations

ALC: Absolute lymphocyte count
NLR: Neutrophil-to-lymphocyte ratio
A/MBC: Advanced or metastatic breast cancer
OS: Overall survival
EMT: Epithelial-to-mesenchymal transition
HER2: Human epidermal growth factor receptor 2
PFS: Progression-free survival
eMRs: Electronic medical records
CMUH: China Medical University Hospital
IQR: Interquartile range
ER: Oestrogen receptor
PR: Progesterone receptor
HR: Hazard ratio
CI: Confidence interval
IT: Information technology
IRB: Institutional Review Board
