# Supplementary material for: Monitoring trends in the absolute lymphocyte count and the neutrophil-to-lymphocyte ratio in patients with breast cancer receiving eribulin
Source: BMC Cancer. 2024 Feb 12;24:195. doi: 10.1186/s12885-024-11923-5 (PMC10860250; doi:10.1186/s12885-024-11923-5)
Supplement: Supplementary file 1 — Additional file 1: Supplementary Figure 1. Overall survival (OS) among patients with increased or decreased trends of ALC, NLR (0: decreasing, 1: non-decreasing). Supplementary Figure 2. Comparing PFS and OS among different groups of patients stratified by ALC trends according to the baseline level. Supplementary Figure 3. Comparing PFS and OS among different groups of patients stratified by NLR baseline and trends. Supplementary Figure 4. Analysis on the association of high(1)/low(0) NLR/ALC with OS stratified by on(1)/off(0) treatment with eribulin. [file 12885_2024_11923_MOESM1_ESM.docx]

**Supplementary**

| ALC | NLR |
| --- | --- |
| 1. 1m vs baseline   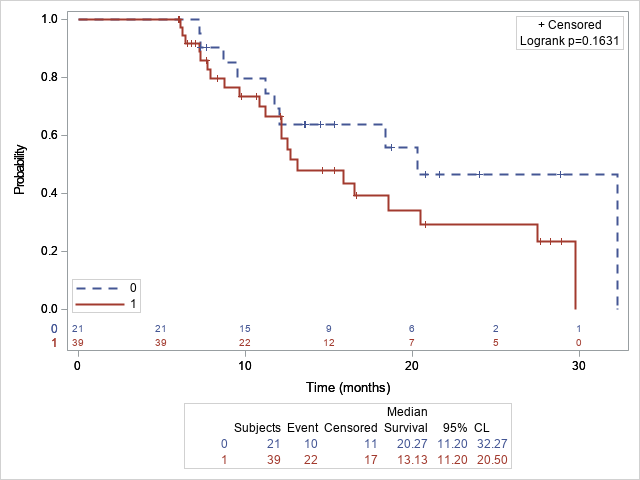 | D. 1m vs baseline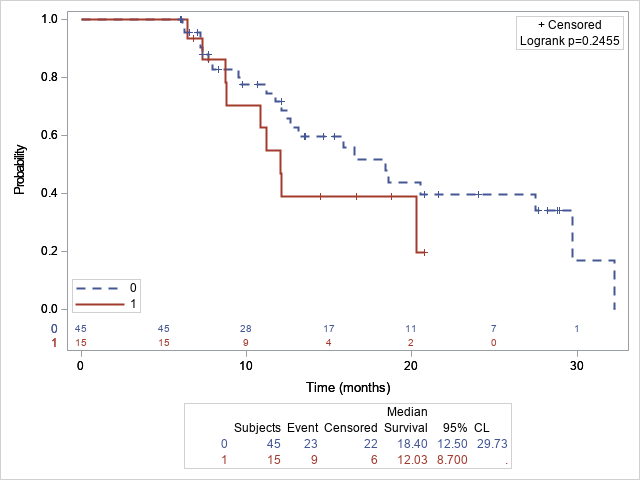 |
| 1. 3m vs baseline   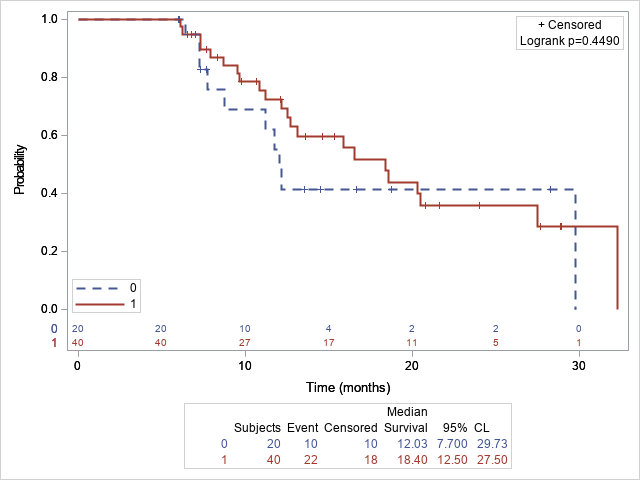 | E. 3m vs baseline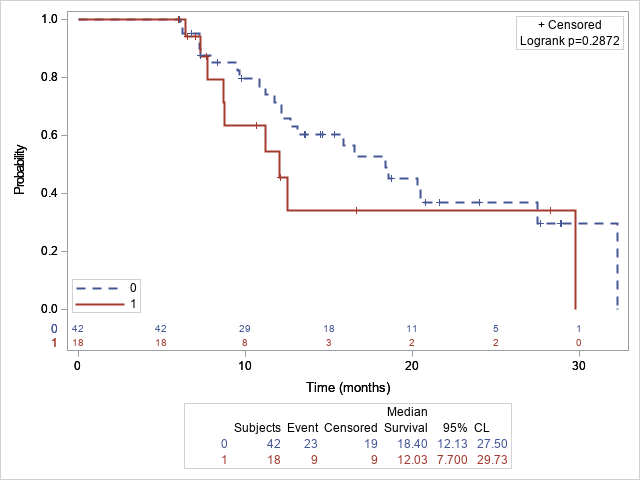 |
| 1. 6m vs baseline   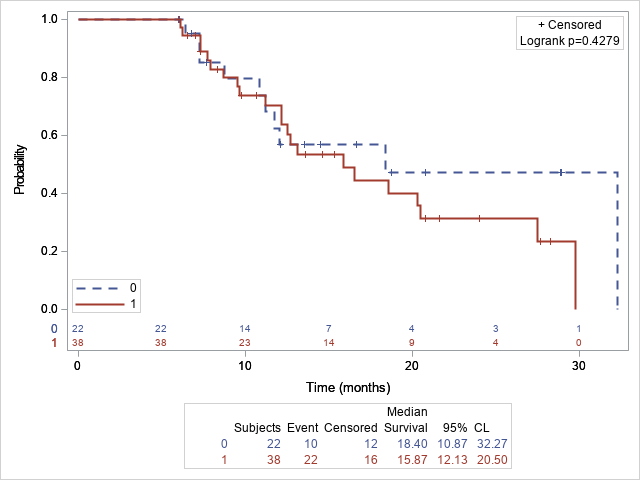 | F. 6m vs baseline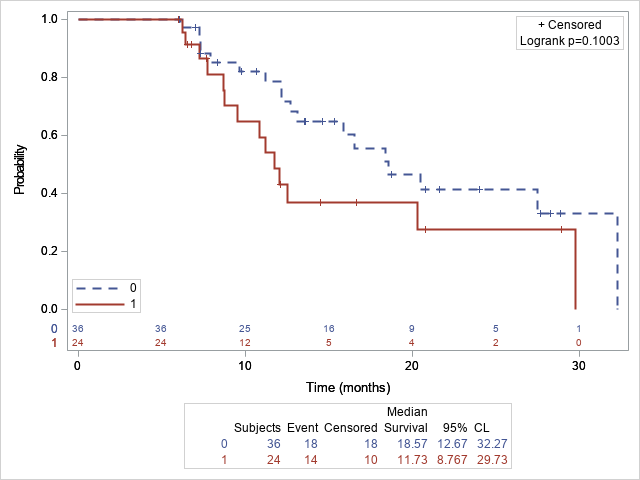 |

ALC= absolute lymphocyte count; NLR=neutrophil to lymphocyte ratio

**Supplementary Figure. 1. Overall survival (OS) among patients with increased or decreased trends of ALC, NLR (0: decreasing, 1: non-decreasing)**

| **PFS** | **OS** |
| --- | --- |
| 1. 1m vs baseline   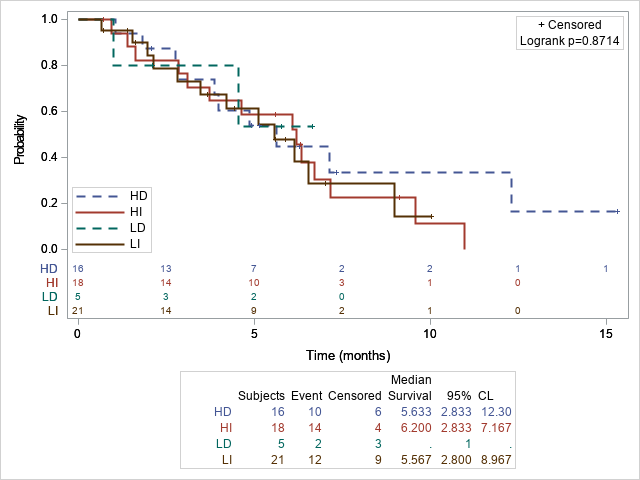 | 1. 1m vs baseline   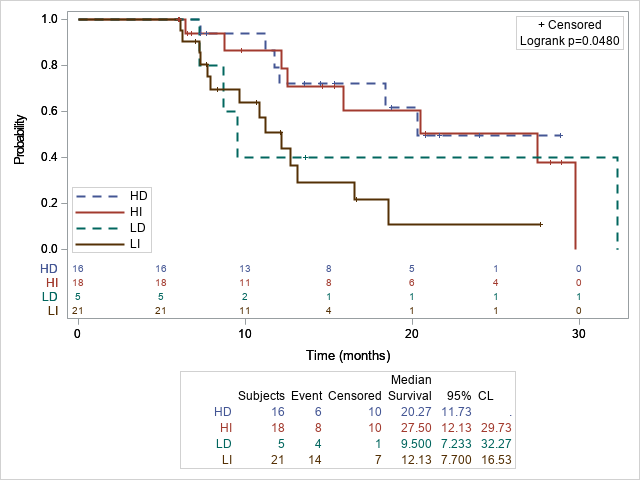 |
| 1. 3m vs baseline   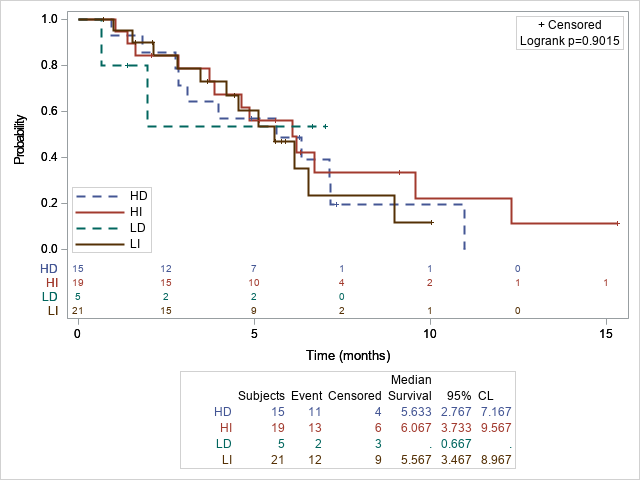 | 1. 3m vs baseline   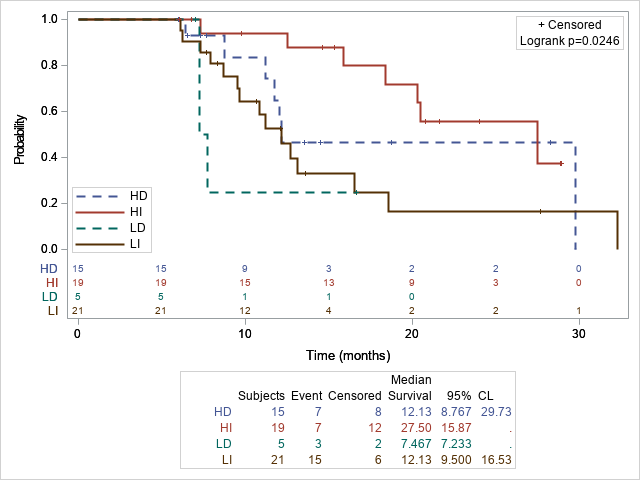 |
| 1. 6m vs baseline   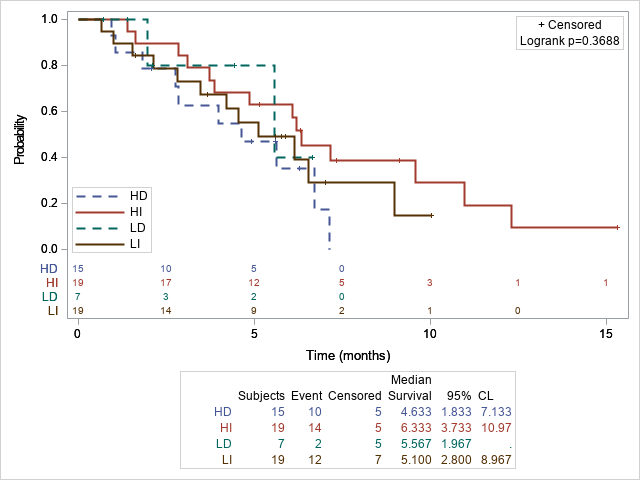 | 1. 6m vs baseline   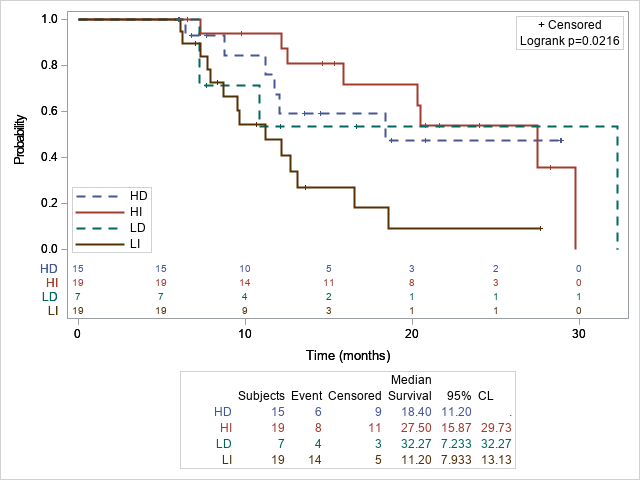 |

HD= Initially high then decreasing; HI= Initially high then increasing; LD= Initially low then decreasing; LI= Initially low then increasing; PFS=Progression-free-survival; OS= Overall survival; ALC=absolute lymphocyte count

**Supplementary Figure 2. Comparing PFS and OS among different groups of patients stratified by ALC trends according to the baseline level.**

| **PFS** | **OS** |
| --- | --- |
| 1. 1m vs baseline | 1. 1m vs baseline |
| 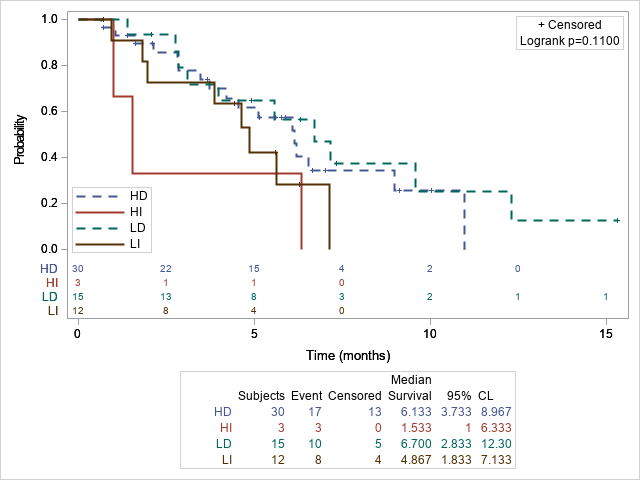 | 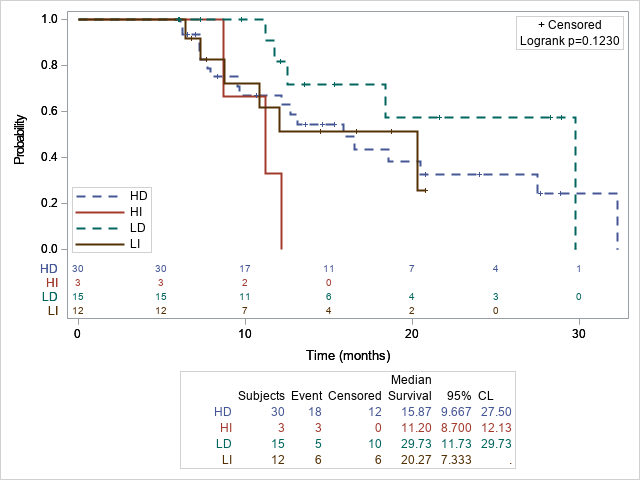 |
| 1. 3m vs baseline | 1. 3m vs baseline |
| 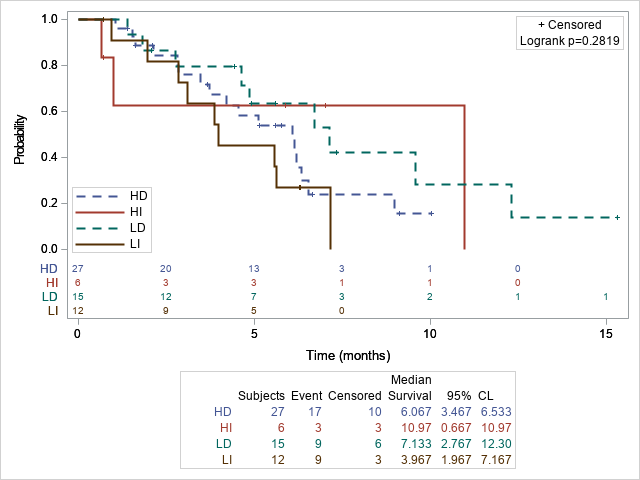 | 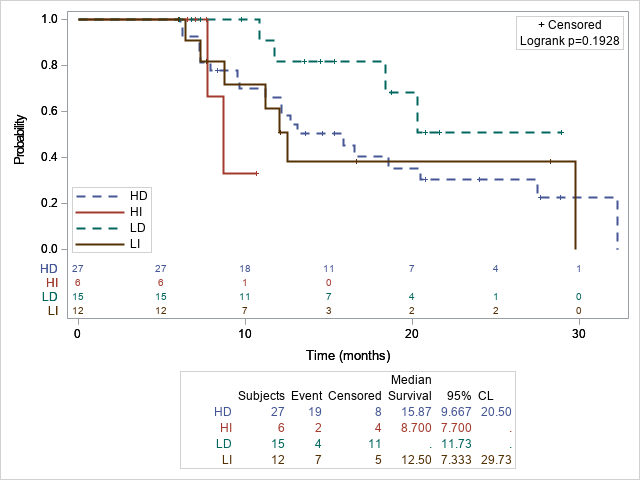 |
| 1. 6m vs baseline | 1. 6m vs baseline |
| 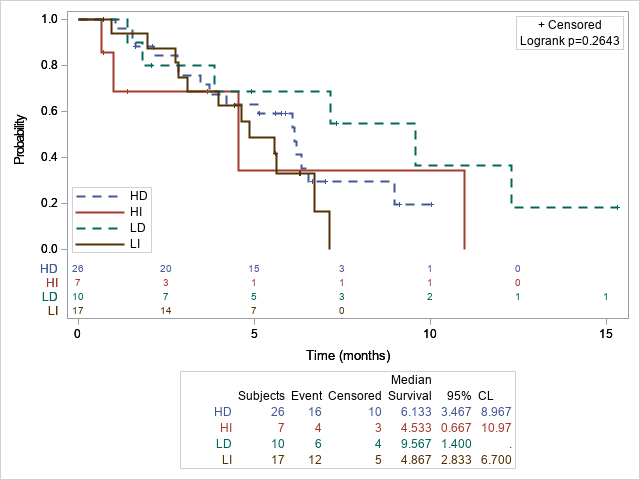 | 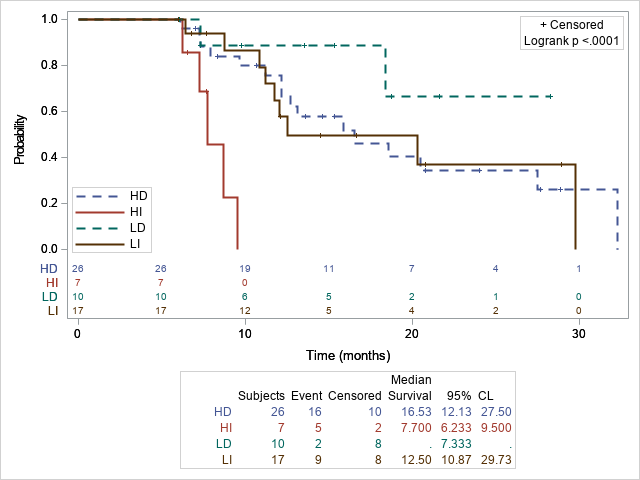 |

HD= Initially high then decreasing; HI= Initially high then increasing; LD= Initially low then decreasing; LI= Initially low then increasing; PFS=Progression-free-survival; OS= Overall survival; NLR=neutrophil to lymphocyte ratio

**Supplementary Figure 3. Comparing PFS and OS among different groups of patients stratified by NLR baseline and trends.**

| ALC | NLR |
| --- | --- |
| At 3 month-test 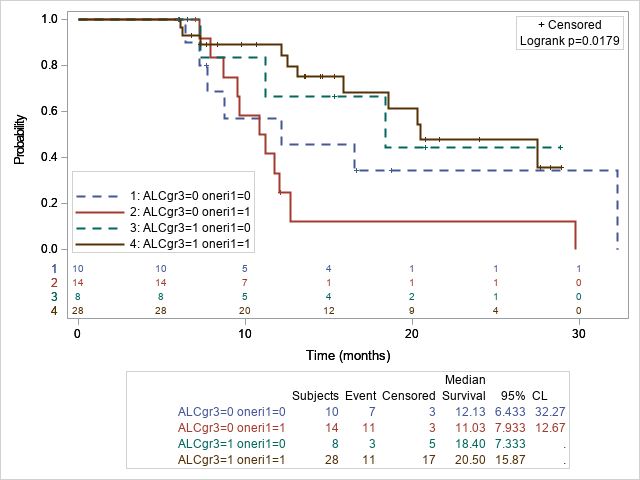 | At 3 month-test 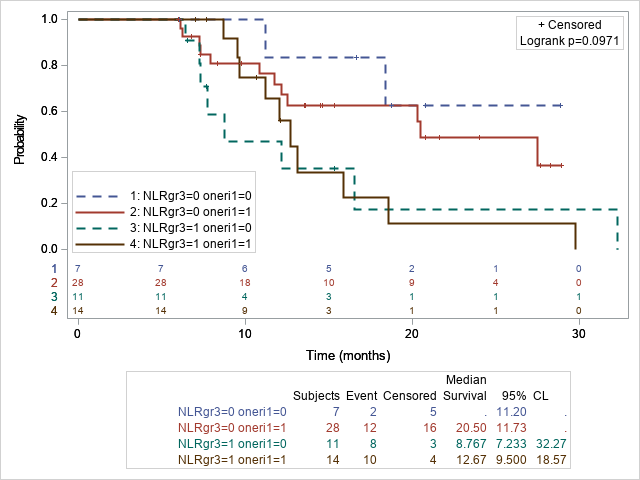 |
| At 6-month-test 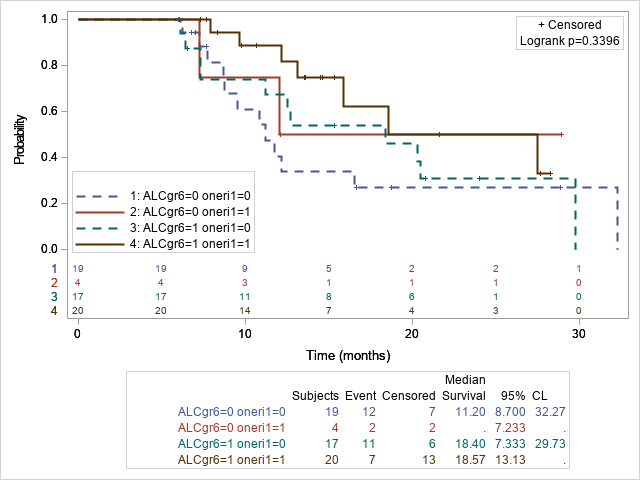 | At 6-momnth test 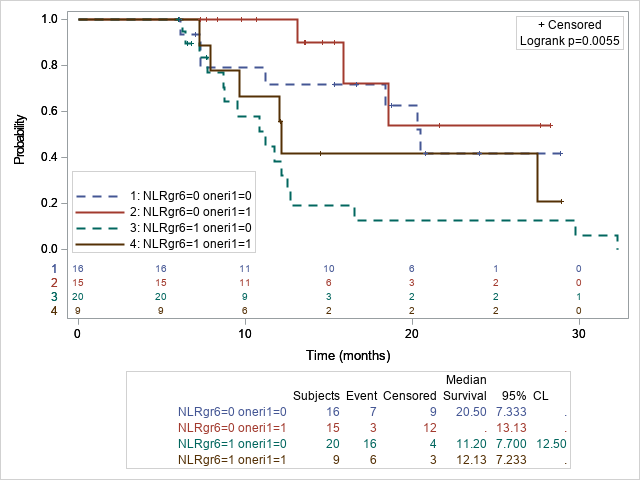 |

ALC= absolute lymphocyte count; NLR=neutrophil to lymphocyte ratio; OS= Overall survival;

ALCgr3=0 Oneri1=0 refers patients with low ALC and off eribulin treatment at 3 month; ALCgr3=0 Oneri1=1 refers patients with low ALC and on eribulin treatment at 3 month; ALCgr3=1 Oneri1=0 refers patients with high ALC and off eribulin treatment at 3 month; ALCgr3=1 Oneri1=1 refers patients with high ALC and on eribulin treatment at 3 month

ALCgr6=0 Oneri1=0 refers patients with low ALC and off eribulin treatment at 6 month; ALCgr6=0 Oneri1=1 refers patients with low ALC and on eribulin treatment at 6 month; ALCgr6=1 Oneri1=0 refers patients with high ALC and off eribulin treatment at 6 month; ALCgr6=1 Oneri1=1 refers patients with high ALC and on eribulin treatment at 6 month

**Supplementary Figure 4: Analysis on the association of high(1)/low(0) NLR/ALC with OS stratified by on(1)/off(0) treatment with eribulin**
